# From Evidence to Insight: An Umbrella Review of Computational Thinking Research Syntheses

**DOI:** 10.3390/jintelligence13120157

**Published:** 2025-12-02

**Authors:** Jin Zhang, Yaxin Wu, Yimin Ning, Yafei Shi

**Affiliations:** 1 Faculty of Education, Henan Normal University, Xinxiang 453007, China; wuyaxin@stu.htu.edu.cn; 2 School of Mathematical Sciences, East China Normal University, Shanghai 200241, China

**Keywords:** computational thinking, systematic review, meta-analysis, umbrella review, pedagogical issues, literature review

## Abstract

This study reviews 33 meta-analyses and systematic reviews on Computational Thinking (CT), focusing on research quality, intervention effectiveness, and content. Quality assessment of included studies was conducted using the AMSTAR 2 tool. The meta-analysis achieved an average score of 10.9 (a total of 16 points), while systematic reviews scored an average of 6.1 (a total of 11 points). The 15 meta-analyses showed diverse intervention strategies. Project-based learning, text-based programming, and game-based learning demonstrate more pronounced effects in terms of effect size and practical outcomes. Curricular integration, robotics programming, and unplugged strategies offered additional value in certain contexts. Gender and disciplinary background were stable moderators, while grade level and educational stage had more conditional effects. Intervention duration, sample size, instructional tools, and assessment methods were also significant moderators in several studies. The 18 systematic reviews used a five-layer framework based on ecological systems theory, covering educational context (microsystem), tools and strategies (mesosystem), social support (exosystem), macro-level characteristics (macrosystem), and CT development (chronosystem). Future research should focus on standardizing meta-analyses, unifying effect size indicators, and strengthening longitudinal studies with cognitive network analysis. Additionally, systematic reviews should improve evidence credibility by integrating textual synthesis and data-driven reasoning to reduce redundancy and homogeneity.

## 1. Introduction

Computational thinking (CT) is a higher-order cognitive skill that has evolved from a specialised area of computer science to become a core part of digital literacy for citizens in the digital age (Wu et al. 2024). Its conceptual origins can be traced back to Seymour Papert’s pioneering work in the 1980s on the influence of computers on children’s thinking, and to Jeannette Wing’s subsequent 2006 article, in which she formalised CT as a universal cognitive skill (Papert 1980; Wing 2006). Wing (2006) defined CT as the application of fundamental computer science concepts to problem-solving, system design, and understanding human behavior. International organizations, including the OECD, the European Commission, and UNESCO, have since integrated CT into global education policies, solidifying its strategic importance (Bers et al. 2022; Bocconi 2016; Vakhabova et al. 2025). While debates over its precise definition continue, a consensus is emerging that CT is a composite thinking system involving cognitive, affective, and social interactions. For the purposes of this study, we draw upon the competency models proposed by Kraiger et al. (1993) and Kurz and Bartram (2002) in order to conceptualise CT as a continuum spanning ‘cognition–behaviour’. This continuum comprises four interrelated levels: (1) ability, representing an individual’s inherent algorithmic logical potential; (2) skills, denoting decomposable, teachable procedural knowledge units; (3) competency, a contextualised, developable construct integrating knowledge, skills and attitudes; and (4) performance, representing the behavioural output of competency in specific tasks that is susceptible to interference from motivational and environmental factors. This conceptual framework provides a theoretical foundation for understanding the multidimensional nature of CT and evaluating its educational effectiveness.

As a composite thinking system, CT exhibits complex characteristics such as situational adaptability, developmental nonlinearity and multidimensional assessment. Firstly, its situational adaptability emphasises that CT is not a rigid set of rules, but rather an individual’s ability to adjust their thinking strategies dynamically according to the context of a specific problem. This concept is deeply rooted in constructivist theory regarding the generation of knowledge through practice (Kafai 2005). Secondly, the non-linear development of CT challenges traditional linear notions of progress by indicating that its growth involves complex, non-sequential interactions between different knowledge structures and skills (Román-González et al. 2018). These characteristics make assessing CT particularly challenging. Finally, single-dimensional measurement tools struggle to capture its full scope. Effective evaluation must therefore integrate competencies, skills, capabilities and behavioural performance, while also accounting for individual motivation and environmental influences (Kanaki et al. 2025).

Researchers have demonstrated numerous effective intervention strategies for developing students’ CT. Countries worldwide are integrating CT into their basic education curricula, from an introduction in elementary school to a deeper study in high school. This makes educational interventions a crucial pathway for enhancing students’ CT (Gutiérrez-Núñez et al. 2022). For example, the UK’s 2014 Computer Science Curriculum Framework made computer science (including CT) a compulsory subject (Passey 2017). The U.S. Department of Education’s Office of Technology, Innovation, and Learning prioritises CT in early childhood education and has systematised CT instruction in the K–12 Computer Science Framework (Ballard and Haroldson 2021). China’s 2017 General High School Information Technology Curriculum Standards were the first to designate CT as a core competency (Li et al. 2024). Intervention practices and empirical research on CT instruction have proliferated rapidly across diverse educational stages and teaching formats. For example, Ye et al. (2023) analysed the effectiveness of integrating CT into mathematics education at primary and secondary level, demonstrating its significant support for student learning. Lin et al. (2021) used technologies such as augmented reality and AIoT to facilitate effective CT training at university level. Cultivating students’ CT enables meaningful engagement, enhances the capacity to manage complex problems systematically, heightens awareness of critical global issues and facilitates tangible contributions to sustainable development.

CT is an emerging, multifaceted competency, yet research on its educational integration remains underdeveloped. Although some meta-analyses and reviews report significant improvements from well-designed interventions (Ye et al. 2023), others suggest CT interventions warrant closer examination (Kite et al. 2019), citing concerns over limited generalizability or transient effects. Furthermore, empirical evidence remains fragmented, with persistent discrepancies regarding the influence of moderating variables, such as intervention duration, sample size, participant demographics, and disciplinary background. These inconsistencies highlight a critical need for more rigorous, multidimensional assessment methods to determine the comprehensiveness and effectiveness of CT research.

To address this gap, this study employs an umbrella review, a high-level systematic research method. Unlike meta-analyses, which often focus on single intervention comparisons, umbrella reviews synthesize existing systematic reviews and meta-analyses to provide the strongest level of evidence (Wohlfart and Wagner 2023). This approach offers a comprehensive synthesis by concentrating on broad themes, thus demonstrating the effects of a wider range of educational interventions on target variables.

In this study, we used the Preferred Reporting Items for Systematic Reviews and Meta-Analyses (PRISMA) protocol to ensure review transparency and reproducibility (Martins de Souza et al. 2024; Page et al. 2021), and the Assessment of Measurement Tools for Systematic Reviews (AMSTAR 2) instrument to comprehensively evaluate study quality (Shea et al. 2017). Additionally, we adopted ecological systems theory as an analytical framework to integrate multi-level factors ranging from direct teaching environments to broader external contexts, thereby offering a more holistic account of the complex mechanisms underlying CT development and enhancing the explanatory rigor and practical implications of this study. The study addresses three research questions:

RQ1. What is the quality of meta-analyses and systematic reviews related to CT, and what overall trends do they reflect?

RQ2. How effective are different types of CT intervention strategies, and what key moderating variables influence their outcomes?

RQ3. How are factors influencing CT development distributed across ecological system levels (micro, meso, exo, macro, chrono)?

## 2. Materials and Methods

**Figure 1:**
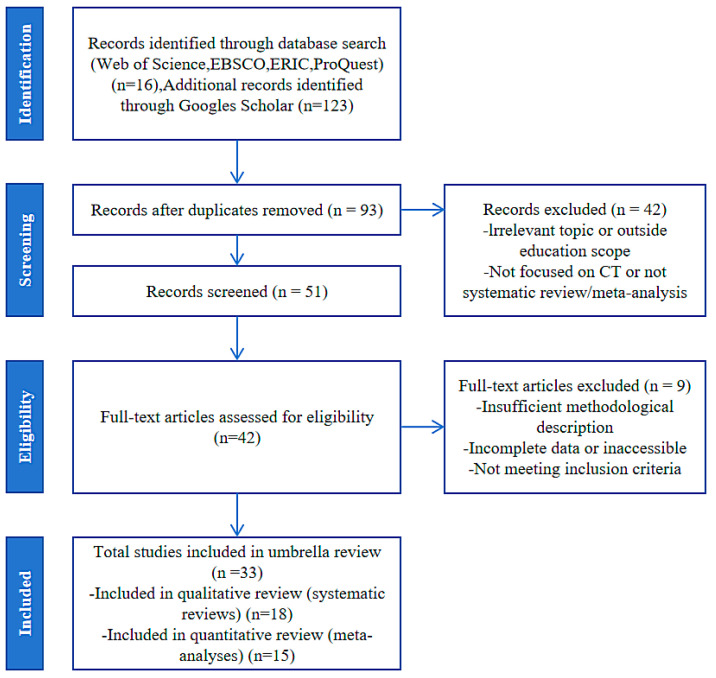
PRISMA flow diagram.

The present umbrella review was conducted and reported in accordance with the Preferred Reporting Items for Systematic Reviews and Meta-Analyses (PRISMA) guidelines (Martins de Souza et al. 2024; Page et al. 2021) (see Figure 1). The study protocol has been prospectively registered with the International Prospective Register of Systematic Reviews (PROSPERO) under the registration number CRD420251059546.

### 2.1. Data Analysis

#### 2.1.1. Data Interpretation

This study used Hedges’ g to compare the effect sizes of all the included meta-analyses. For meta-analyses that used different equations to calculate effects (e.g., SMD or Cohen’s d), we applied the corresponding conversion method. Specifically, we converted Cohen’s d to Hedges’ g using formula (1). When sample sizes were large (e.g., >100), Hedges’ g and Cohen’s d were ‘virtually identical’. Meanwhile, SMD (Standardised Mean Difference) is essentially equivalent to Hedges’ g (as defined by Borenstein et al. (2009): SMD = Hedges’ g, or ‘adjusted d’). Hedges’ g results are categorised into three levels: small effect (approximately 0.2), moderate effect (approximately 0.5) and large effect (approximately 0.8).

(1) $$g=d\times (1-\frac{3}{4(n_{1}+n_{2})-9})$$

#### 2.1.2. Quality Evaluation

The methodological quality of the included literature was systematically evaluated using the A Measurement Tool to Assess Systematic Reviews (AMSTAR 2) instrument. AMSTAR 2 consists of 16 checklist items, each scored as “Yes” (1 point), “Partial Yes” (0.5 points), or “No” (0 points), and is designed to identify potential risk of bias in systematic reviews and meta-analyses. Overall quality was categorized into three levels based on the percentage of the total possible score (16 points): high quality (≥80%), moderate quality (40–79%), and low quality (<40%) (Shea et al. 2017). In addition, a simplified evaluation was conducted with reference to the original 11-item AMSTAR tool, where a total score of 0–4 indicates low quality, 5–8 indicates moderate quality, and 9–11 indicates high quality. Given that certain items in the AMSTAR 2 checklist (e.g., Items A11–A15) are not applicable to systematic reviews that do not conduct meta-analyses, this study included only 11 items that are universally relevant to all systematic reviews for the purpose of quality evaluation.

#### 2.1.3. Evaluation of Heterogeneity Between Studies

This study employed meta-regression analysis to examine the potential influence of methodological quality on reported effect sizes in meta-analyses. We utilized AMSTAR 2 assessment scores as a measure of study quality, converting these scores into two categories—high-quality and low-quality—using the median as the cutoff point. The effect size was measured using Hedges’ g. All analyses were conducted in R 4.2.1 (R Core Team 2021) using the “metafor” package for data processing. For model estimation, inter-study variance (τ^2^) was estimated using restricted maximum likelihood (REML), following (Viechtbauer 2010) recommendation for random-effects meta-regression to robustly address inter-study heterogeneity and enhance model fit precision. Concurrently, we assessed overall heterogeneity using the I^2^ statistic and integrated both within-study variation (sampling error) and between-study heterogeneity within the meta-regression. To ensure robustness, sensitivity analyses were conducted by excluding extreme effect values (the three studies at each end of the effect size distribution) and re-running the grouped meta-regression analysis on the filtered dataset.

### 2.2. Search Strategy

This study focuses on evaluating the methodological rigor and research landscape of existing evidence synthesis on CT in the field of education. A comprehensive literature search was conducted across four authoritative databases: Web of Science, EBSCO, ERIC, and ProQuest, supplemented by additional searches through Google Scholar. The search strategy employed the keywords “computational thinking”, “systematic review”, and “meta-analysis”, combined using Boolean operators “AND” and “OR” to ensure both thematic specificity and broad coverage. The inclusion criteria were restricted to peer-reviewed journal articles, specifically systematic reviews and meta-analyses, to ensure the quality and validity of the included literature. Given that the first high-quality review in the CT field was conducted by Hsu et al. (2018), the publication window was restricted to articles published between January 2018 and December 2024. An initial total of 239 records was retrieved—116 from formal databases and 123 from Google Scholar. The screening process followed the PRISMA flow diagram. After removing duplicates, 93 unique records remained. Title and abstract screening excluded 42 studies that did not meet the research scope or were of inappropriate document types. The remaining 51 studies were assessed for full-text eligibility. Ultimately, 9 articles were excluded due to incomplete methodology or failure to meet inclusion criteria. For instance, when two or more meta-analyses studied an identical topic, we excluded literature with low AMSTAR 2 scores, imprecise estimates, or meta-analyses with limited sample sizes to avoid overlaps. A total of 33 studies were included in the final umbrella review, comprising 18 systematic reviews and 15 meta-analyses.

To ensure objectivity and consistency in the literature screening and data extraction process, a double independent coding method was employed to extract key information from the included studies. The extracted elements included the source database, types of interventions, effect sizes, and moderating variables. During the initial coding phase, two researchers independently coded the same sample of studies. Upon completion, the results were compared to evaluate agreement. To assess inter-coder reliability, Cohen’s Kappa coefficient was calculated. In general, Kappa values between 0.61 and 0.80 are considered to indicate substantial agreement, while values above 0.81 reflect almost perfect agreement. In this study, all primary coding dimensions achieved Cohen’s Kappa values exceeding 0.75, suggesting a high level of inter-coder agreement. For coding entries where discrepancies occurred, a third reviewer was consulted, and final consensus was reached through discussion. This process ensured the scientific rigor and reliability of the review findings.

### 2.3. Selection and Exclusion Criteria

To ensure the scientific validity and relevance of the included literature, this study applied clearly defined inclusion and exclusion criteria. Studies were included if they focused on CT and its application within the field of education; employed a systematic review or meta-analysis methodology; targeted student or teacher populations across educational levels, including K-12, higher education, and teacher education; were published in English in peer-reviewed journals; and had a publication date no earlier than 2006. Studies were excluded if they did not address core aspects of CT (e.g., focused only on general programming or technology use); did not adopt systematic review or meta-analysis methodologies (e.g., narrative reviews, opinion articles, or case studies); targeted non-educational populations such as those in medical education or informal learning contexts; lacked clear methodological transparency (e.g., missing descriptions of search strategies, screening procedures, or data analysis); had inaccessible full texts or severely incomplete data; or were duplicate publications, in which case only the most complete or most recently published version was retained. The application of these criteria enhanced the systematicity, credibility, and authoritativeness of this umbrella review.

## 3. Results

### 3.1. Quality and Overall Trends of Included Studies

#### 3.1.1. Descriptive Characteristics of Included Studies

**Figure 2:**
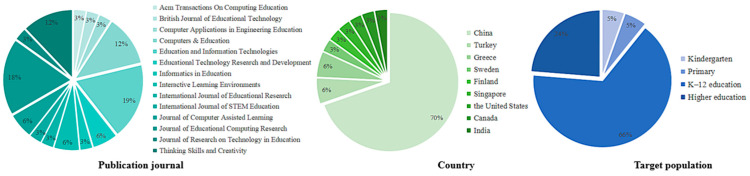
Descriptive characteristics.

To comprehensively understand the basic characteristics of the included literature, this study conducted a statistical classification of all studies that met the inclusion criteria, focusing primarily on publication journals, target populations, and country distribution (see Figure 2). Overall, systematic reviews and meta-analyses in the field of CT have shown a gradual increase since 2006, with a notable surge over the past five years. Systematic reviews were predominantly published between 2018 and 2024, indicating sustained and widespread academic attention to CT within the education sector. Similarly, meta-analyses were primarily concentrated after 2020, reflecting a growing demand for quantitative synthesis as empirical research on CT continues to accumulate.

In terms of journal distribution, the included studies were predominantly published in a small number of Q1 journals in the fields of educational technology and learning sciences. Both the *Journal of Educational Computing Research* and *Education and Information Technologies* each published six systematic review or meta-analysis articles, indicating a relatively high level of attention to structured evidence synthesis in these outlets. *Computers & Education* and *Thinking Skills and Creativity* also contributed four related studies each, maintaining notable academic activity in areas such as educational technology, learning outcomes, and higher-order thinking. Overall, the concentration of studies in a small number of core journals may reflect certain commonalities in their thematic orientations. On the one hand, these journals tend to publish research that is methodologically rigorous, structurally well-organized, and closely connected to instructional or learning contexts; on the other hand, the inherent relevance of computer technology to themes such as technological applications, knowledge transfer, and cognitive development naturally makes it a frequent topic in interdisciplinary research. Taken together, this distribution pattern highlights the key areas of scholarly focus in the field and offers useful reference points for future researchers in terms of journal selection and research design.

In terms of country distribution, the affiliations of the first authors of the included studies reveal significant disparities. Meta-analyses were predominantly led by authors based in China, which contributed 14 studies (accounting for 63.6% of all meta-analyses). Other countries, such as the United States, Singapore, and Finland, showed sporadic contributions. In contrast, the systematic reviews exhibited a more diverse international representation. In addition to China (which accounted for 45.5%), authors from Canada, India, Turkey, Greece, and Sweden independently contributed publications, with Greece alone contributing two studies. The regional distribution of included studies indicates a strong focus on the research on topics related to CT, primarily in the context of China, while other regions remain underrepresented.

In terms of target population, current CT-related review studies demonstrate a distribution pattern characterized by a strong focus on K–12 education, moderate extension into higher education, and limited attention to early childhood and primary levels. Among the included studies, 25 explicitly targeted K–12 students, indicating that this educational stage has become the central arena for CT instructional interventions and assessment research. In addition, nine studies focused on higher education, particularly undergraduate and graduate students, with an emphasis on teacher education, engineering, and computing-related disciplines. These studies reflect the expanded application of CT in specialized and advanced education contexts. However, studies targeting kindergarten and primary-level learners remain scarce, with only two reviews addressing each group. This highlights a significant research gap in understanding CT development among younger learners. Furthermore, some studies employed broad or ambiguous labels such as K–9, or failed to specify target populations altogether-for example, Zhang et al. (2024b)—indicating inconsistencies in population reporting and classification within current review literature, which warrants greater methodological standardization in future studies. Overall, this distribution reflects the practical focus of current research and provides a directional basis for future vertical expansion of CT education and the promotion of lifespan-inclusive research.

In terms of sample size, meta-analytic studies generally involved large-scale datasets, with some studies analyzing data from over 10,000 participants. For instance, Sun et al. (2021) synthesized data from more than 11,000 individuals, demonstrating the extensive empirical basis supporting their findings. In contrast, systematic reviews tend to emphasize thematic synthesis of research content and methodologies, resulting in greater heterogeneity in sample sizes across included studies. Regarding research design, most of the included literature was based on quantitative studies, such as intervention experiments and quasi-experimental designs. However, a number of studies employed mixed-methods approaches that integrated qualitative and quantitative evidence-notably, Yin et al. (2024) and Yeni et al. (2024). This methodological diversity enriches the analytical depth of CT education research by uncovering its underlying mechanisms and practical effectiveness from multiple perspectives.

#### 3.1.2. Quality Evaluation of Included Studies

This study assessed the methodological quality of the included meta-analyses. For the 15 included meta-analyses (see Appendix A Table A1), AMSTAR 2 scores ranged from 10.0 to 13.5, with an average score of 10.9. Among these, 2 studies were rated as high quality, and 13 studies as moderate quality, with no studies falling into the low-quality category. While they demonstrated high adherence to standard procedures in foundational steps such as literature screening and data extraction, deficiencies in key areas related to reducing bias and ensuring transparency persisted. Firstly, most meta-analyses did not report PROSPERO registration (Item A2) or clarify whether their methods were pre-specified prior to implementation, potentially increasing the risk of selective reporting bias. Secondly, regarding the independent execution of study selection and data extraction (Items A5 and A6), many studies did not specify whether two reviewers were involved independently or how consensus was reached, undermining the transparency and reliability of the review process. Moreover, key reporting elements such as lists of excluded studies and justifications for exclusion (Item A7) were frequently omitted, reducing overall transparency. Similarly, funding sources for included studies (Item A10) and conflict of interest disclosures (Item A16) were rarely reported, limiting the ability to assess potential financial or author-related biases. In terms of statistical analysis, some studies did not adequately describe the methods used for effect size synthesis or heterogeneity adjustments (Item A11), and did not fully account for the risk of bias in the interpretation of their results (Items A9, A12, A13).

Among the 18 included systematic reviews (see Appendix A Table A2), AMSTAR 2 scores ranged from 3.5 to 8.5, with an average score of 6.11. According to the scoring criteria, 15 studies were rated as moderate quality, 3 studies as low quality, and none reached the high-quality threshold. The results of the quality assessment of systematic reviews further highlight the common methodological challenges faced in evidence synthesis research in this field, particularly with regard to ensuring transparency of processes and controlling for selective reporting bias. While most reviews performed relatively well in terms of developing literature search strategies (Item A4) and reporting study characteristics (Item A8), notable weaknesses were observed in areas related to transparency of study design and bias control. Specifically, none of the reviews employed the PICO framework (Item A1) to structure the research question, nor did they report protocol registration (Item A2), indicating a lack of systematic planning in the early research stages and raising the risk of selective reporting bias. Regarding the execution of study selection and data extraction (Items A5 and A6), most reviews did not clarify whether independent double coding was conducted, reducing the reproducibility and reliability of the review process. In terms of reporting transparency, the majority of reviews failed to meet AMSTAR 2 criteria concerning the provision of an exclusion list with justifications (Item A7), reporting of funding sources for included studies (Item A10), and disclosure of potential conflicts of interest by the authors (Item A16).

**Figure 3:**
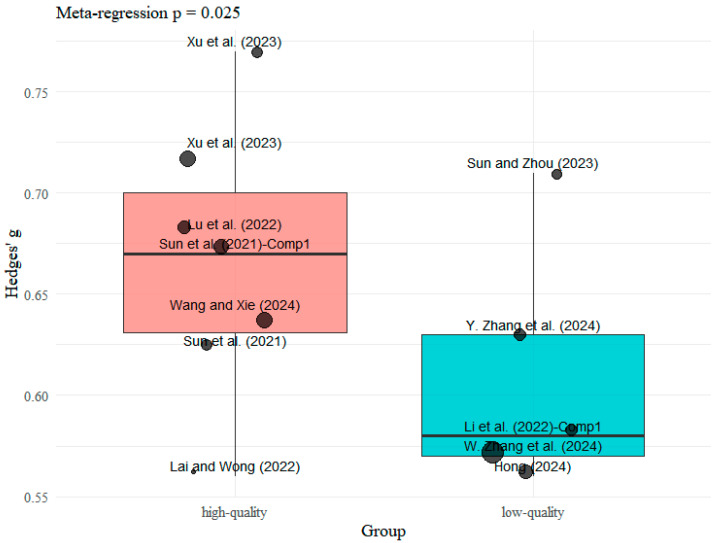
Meta-regression: effect size by study quality (Fidai et al. (2020); Sun et al. (2021); Zhang et al. (2021); Merino-Armero et al. (2022); Li et al. (2022); Lai and Wong (2022); Sun and Zhou (2023); Lu et al. (2022); Hong (2024); Xu et al. (2023); Xu et al. (2023); Wang et al. (2024); Zhang et al. (2024b); Wang and Xie (2024); Zhang et al. (2024a)).

Meta-regression analysis revealed a trend toward a positive association between the methodological quality of meta-analyses (AMSTAR 2 score) and effect size (Hedges’ g). Analysis of all 18 studies indicated that this relationship did not reach statistical significance (regression coefficient = 0.009, 95% CI [–0.058, 0.076], *p* = 0.798). Concurrently, significant heterogeneity existed among studies (I^2^ = 82.4%, *p* < 0.001), indicating that results may be influenced by individual extreme effect sizes, potentially obscuring the true association trend (Alene et al. 2024; Camargo Salamanca et al. 2024; Samdrup et al. 2023). To assess the robustness of regression results and investigate reasons for non-significance, we conducted a sensitivity analysis by excluding the three studies with the smallest and largest effect sizes. As shown in Figure 3, after excluding outliers, a significant positive correlation emerged between quality scores and effect sizes (regression coefficient = 0.091, 95% CI [0.011, 0.170], *p* = 0.025). Specifically, the high-quality group (*n* = 7, mean quality score 11.57) had a mean effect size of 0.666, significantly higher than the 0.610 observed in the low-quality group (*n* = 5, mean quality score 10.00). Furthermore, model heterogeneity completely disappeared after excluding outliers (I^2^ = 0%), indicating that quality score grouping accounted for all between-study variation. These findings suggest that study quality may serve as a positive moderator of effect size. Although the association strength is relatively modest and sensitive to outliers, the sensitivity analysis results support the conclusion that study quality exerts a genuine moderating effect on effect size.

#### 3.1.3. Statistical Analysis of Overall Research Trends

In order to visually present the temporal distribution of CT research in systematic reviews and meta-analyses, two Gantt charts were generated to display the publication timelines of the included studies in each category.

**Figure 4:**
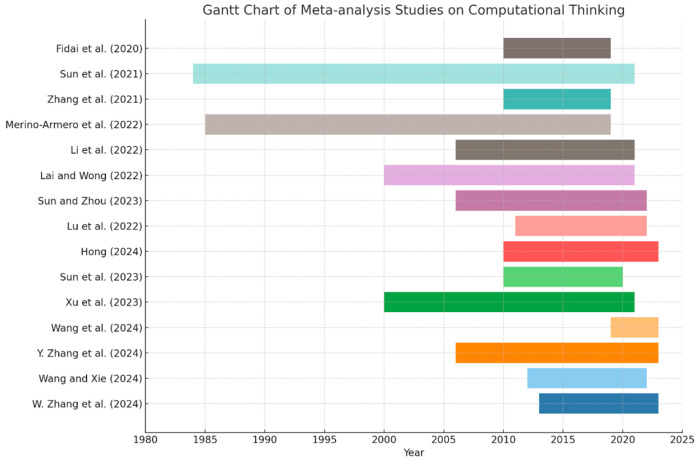
Meta-analysis studies Gantt chart (Fidai et al. (2020); Sun et al. (2021); Zhang et al. (2021); Merino-Armero et al. (2022); Li et al. (2022); Lai and Wong (2022); Sun and Zhou (2023); Lu et al. (2022); Hong (2024); Xu et al. (2023); Xu et al. (2023); Wang et al. (2024); Zhang et al. (2024b); Wang and Xie (2024); Zhang et al. (2024a)).

As shown in Figure 4, meta-analytic research in the field of CT exhibits an evolutionary trajectory characterized by a concentrated onset, rapid development, and gradual updating. Early studies, such as those by Sun et al. (2021) and Merino-Armero et al. (2022), featured notably broad time spans, reflecting systematic retrospection of CT’s developmental trajectory. These foundational works not only examined intervention effectiveness but also helped establish an early evidence base for CT education, laying the groundwork for subsequent inquiries. Since 2010, there has been a marked increase in the number of meta-analyses, with a significant concentration of publications between 2020 and 2023, signaling a peak period of scholarly activity. Studies from this phase generally focused on data collected post-2000, showing greater thematic concentration, with research objectives increasingly oriented toward comparing intervention strategies and analyzing moderating variables. This shift reflects the growing role of meta-analysis as a core methodological tool in evaluating the effectiveness of CT-related educational interventions. In 2024, the number of published meta-analyses rose sharply, indicating an accelerating update cycle. However, many of these recent studies had shorter temporal spans, typically between 5 to 10 years—for example, the study by Wang et al. (2024) covered a period of less than five years. This trend suggests a rapid response to emerging intervention trends, but also highlights the tension between timely updates and long-term evidence accumulation. Overall, the temporal distribution of CT-related meta-analyses reveals a pattern of “deep foundation followed by rapid iteration.” While early studies focused on building the evidence base, more recent work emphasizes methodological diversity and evidence integration. As CT research continues to expand and empirical literature becomes more abundant, future meta-analyses should aim to enhance update mechanisms and strengthen the capacity to assess both long-term and short-term intervention effects, thereby advancing the application of evidence-based practice in CT education.

**Figure 5:**
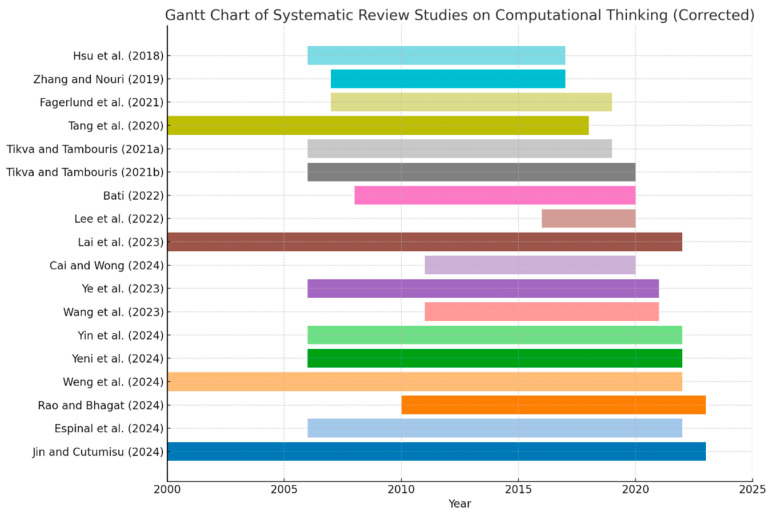
Systematic review studies Gantt chart (Hsu et al. (2018); Zhang and Nouri (2019); Fagerlund et al. (2021); Tang et al. (2020); Tikva and Tambouris (2021a); Tikva and Tambouris (2021b); Bati (2022); Lee et al. (2022); Lai et al. (2023); Cai and Wong (2024); Ye et al. (2023); Wang et al. (2023); Yin et al. (2024); Yeni et al. (2024); Weng et al. (2024); Rao and Bhagat (2024); Espinal et al. (2024); Jin and Cutumisu (2024)).

As illustrated in Figure 5, systematic reviews in the field of CT demonstrate an evolutionary trend of fragmented exploration, rapid accumulation, and broad adoption. The earliest systematic reviews emerged around 2018, such as the influential work by Hsu et al. (2018), and were generally characterized by shorter time spans, reflecting the exploratory nature and theoretical framing efforts during the initial stages of CT research. Since 2020, the number of systematic reviews has grown rapidly, culminating in a publication surge in 2024. A series of high-profile reviews—such as those by Espinal et al. (2024); Yeni et al. (2024), and Cai and Wong (2024)—have marked a transition of systematic review methodology from peripheral to mainstream within the CT research community. In terms of temporal scope, most reviews began their analyses from around 2005–2010. However, some studies, such as Lai et al. (2023) and Jin and Cutumisu (2024), extended their review period back to the 1990s, providing a systematic trace of policy evolution and research traditions in CT education. While such reviews offer a valuable macro-level perspective, they tend to have limited update capacity in addressing the most recent developments. Regarding duration structure, the majority of reviews covered a span of 10 to 20 years, a length that enables the capture of changes in educational policies, shifts in intervention formats, and trends in disciplinary integration. This reflects the responsiveness and adaptability of contemporary systematic reviews to emerging issues in CT education. Overall, systematic reviews in the CT domain have struck a balance between historical retrospection and current responsiveness. On one hand, they accumulate long-term evidence to illuminate the sustained impact and developmental trajectories of CT interventions; on the other, they quickly synthesize the most recent findings to inform educational practices and policy adjustments. These dual functions render systematic reviews an increasingly indispensable tool for evidence integration in the field of CT.

### 3.2. Umbrella Review of Meta-Analyses

#### 3.2.1. Intervention Strategies in CT Education

**Figure 6:**
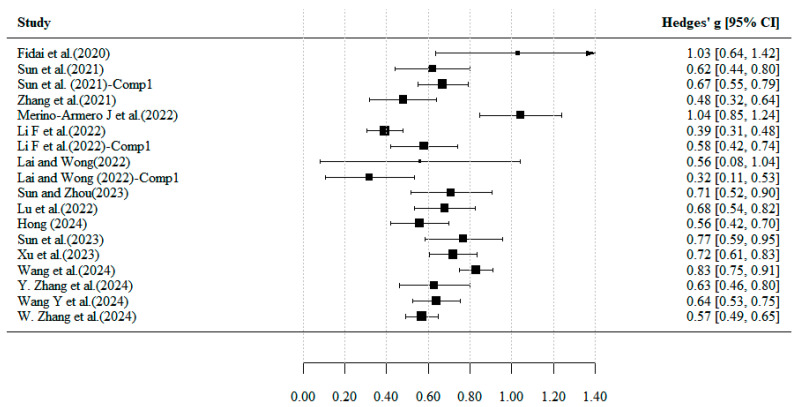
Forest plot of meta-analyses (Fidai et al. (2020); Sun et al. (2021); Zhang et al. (2021); Merino-Armero et al. (2022); Li et al. (2022); Lai and Wong (2022); Sun and Zhou (2023); Lu et al. (2022); Hong (2024); Sun et al. (2023); Xu et al. (2023); Wang et al. (2024); Zhang et al. (2024b); Wang and Xie (2024); Zhang et al. (2024a)).

To gain a comprehensive understanding of the overall effectiveness of CT-related educational interventions, this study systematically reviewed and compared the intervention strategies reported in the included systematic reviews and meta-analyses. During analysis, it was noted that studies employed varied measures of effect size, including SMD, Cohen’s d, and Hedges’ g. To standardize comparisons across all included meta-analyses, we exclusively used Hedges’ g, applying corresponding conversion methods for those studies originally reporting SMD or Cohen’s d (see Figure 6). The results indicate that current CT interventions exhibit a high degree of diversity, with studies employing a range of intervention strategies including project-based learning, programming practice, gamified instruction, and unplugged activities (See Appendix A Table A3). To further highlight the variations in intervention characteristics and associated effect sizes, the analysis is organized into three dimensions: instructional models, programming formats, and learning modalities.

Instructional models identified in the included studies can be broadly categorized into three types: project-based learning, curriculum-based instruction, and experiential learning. Among these, project-based learning demonstrated the highest intervention effectiveness. For instance, Fidai et al. (2020) reported a large effect size of g = 1.030 (95% CI [0.63, 1.42]) based on CT instruction using Arduino projects. Similarly, a meta-analysis by Wang et al. (2024) on integrated experiential project-based interventions reported an effect size of g = 0.83 (95% CI [0.73, 0.89]). Both studies highlight the critical role of task-driven learning, deep engagement, and multi-step problem solving in fostering the development of CT skills.

Curriculum-based instruction also showed moderately high intervention effects. Merino-Armero et al. (2022) found that general CT-oriented instructional curricula achieved an effect size of g = 1.044 (95% CI [0.398, 0.646]). However, the impact of curriculum-based interventions exhibited considerable heterogeneity, influenced by varying learning contexts and implementation differences across instructors.

Within the domain of experiential learning, robot-assisted learning emerged as a representative approach. Three studies focused on this strategy, emphasizing physical interaction and tangible engagement. For example, Zhang et al. (2021) reported an effect size of g = 0.480 (95% CI [0.32, 0.64]) in a systematic evaluation of educational robotics; Hong (2024) found g = 0.558 (95% CI [0.419, 0.697]) for a robot-based instructional program; and Wang and Xie (2024) reported g = 0.643 (95% CI [0.528, 0.757]) for a robotics-integrated learning design. All three studies fell within the moderate effect size range, suggesting that robot-supported instruction can enhance learning motivation and perceptual engagement through hands-on manipulation and visual feedback. However, further instructional design optimization is needed to enhance learning outcomes in terms of logical structuring and programming proficiency. The effectiveness of such interventions may be moderated by factors such as task complexity, depth of interaction with the robotics platform, and instructor scaffolding strategies.

In terms of programming formats, interventions were broadly categorized into three types: text-based programming, robot-based programming, and unplugged programming. Text-based programming demonstrated the largest effect sizes. Sun and Zhou (2023) reported an effect size of g = 0.71 (95% CI [0.51, 0.90]) in an empirical study focused on CT instruction using text-based environments such as Python. Similarly, a meta-analysis by Sun et al. (2021) on individual programming practices yielded an effect size of g = 0.601 (95% CI [0.442, 0.801]). These findings highlight the positive impact of structured syntax training and command-based logic on the development of CT competencies.

Robot-based programming yielded slightly lower effect sizes. For example, Zhang et al. (2021) reported g = 0.480, while Wang and Xie (2024) reported g = 0.643, suggesting that this approach may be particularly suitable for novice learners and perception-driven tasks, where hands-on interaction enhances engagement but may not fully develop abstract programming logic. Unplugged programming exhibited polarized effects. In Li et al. (2022) traditional unplugged activities produced a modest effect size of g = 0.392 (95% CI [0.308, 0.475]), whereas the study by Zhang et al. (2024b), which incorporated elements of logical reasoning, reported a substantially higher effect of g = 0.631 (95% CI [0.463, 0.799]). These findings suggest that the effectiveness of unplugged programming is highly contingent on the cognitive complexity and logical structure embedded in the task design.

From the perspective of learning modalities, interventions were primarily categorized into individual learning, collaborative learning, and game-based learning. Individual learning yielded the lowest effect size among all modalities. For example, Lai and Wong (2022) reported an effect size of g = 0.316 for individual problem-solving tasks, suggesting that while solitary practice has a positive impact on CT development, it may offer limited cognitive activation and strategy transfer. In contrast, the same study found that collaborative problem solving produced a higher effect size of g = 0.562, indicating that activities involving problem decomposition, information exchange, and peer debugging facilitate more effective construction of systematic thinking and debugging strategies. Additional support for the benefits of collaborative learning was provided by Weng et al. (2024), who reported an effect size of g = 0.52 for collaborative programming, further validating the reliable advantages of cooperative interventions in enhancing CT skills. Game-based learning showed the largest effect size among those examined. Xu et al. (2023) reported an effect size of g = 0.766 (95% CI [0.580, 0.951]) for an educational game intervention, while Lu et al. (2022) found a similar effect of g = 0.677 (95% CI [0.532, 0.821]) for general gamification strategies. These approaches significantly enhanced student motivation by embedding feedback systems and task mechanics, and promoted critical CT processes such as strategic decision-making, process planning, and outcome validation.

In summary, different intervention strategies exhibit clearly stratified characteristics in terms of effect size, theoretical mechanisms, and learning adaptability. Among them, project-based instruction, text-based programming, and game-based learning emerged as particularly prominent approaches, with their notable effectiveness likely stemming from a combination of factors such as clear structure, explicit feedback, and motivational engagement. In contrast, curriculum-based instruction and robot-assisted learning demonstrated broader applicability but require further optimization in terms of structural depth and cognitive challenge. Unplugged strategies and collaborative learning, while yielding more variable results, serve as effective complements in specific learning contexts, and their success depends on learner characteristics and task complexity.

#### 3.2.2. Moderating Variables in CT Interventions

A total of 17 records reporting empirical analyses of moderating variables affecting the effectiveness of CT interventions were identified and categorized into four major types: learner characteristics, intervention design, instructional tools, and assessment methods. For each moderating factor, statistical indicators such as Q-values and *p*-values were extracted to enhance the interpretation and evaluation of their moderating effects.

- Learner characteristics

Learner characteristics have been widely examined as potential moderating variables to explore individual differences in the effectiveness of CT interventions. The key subdimensions include grade level, subject background, gender and educational level. Overall, the significance and consistency of moderating effects across studies were found to be variable and context-dependent. Grade level was one of the most frequently examined moderators, though findings were inconsistent. For example, Fidai et al. (2020) found no significant moderating effects of grade level across three CT subdimensions: CT concepts (Q = 1.742, *p* = 0.187), CT practices (Q = 0.07, *p* = 0.97), and CT perspectives (Q = 1.29, *p* = 0.26). Similarly, Hong (2024) conducted subgroup analyses of educational robotics and game-based interventions and also found no significant moderation by grade level (Chi^2^ = 3.187, *p* = 0.364), suggesting that in robot-based CT interventions, grade may not be a key determinant. In contrast, Sun et al. (2023) identified grade level as a significant moderator in the context of game-based CT interventions (Q = 10.963, *p* = 0.012), indicating that the role of grade may depend on the intervention format.

Compared to grade level, subject background exhibited more consistent moderating effects. For instance, Sun et al. (2021) reported that subject area significantly moderated the effects of programming-based interventions (Q = 12.360, *p* = 0.045), suggesting that students from different disciplinary backgrounds may vary in logical structuring, problem decomposition, and strategy transfer, thereby influencing CT development. Some meta-analyses, such as Zhang et al. (2024a), reported intervention effect sizes by educational stage but did not test for the overall moderating significance of this variable.

Gender was also found to be a significant moderator in certain studies. For example, Zhang et al. (2021) identified a highly significant gender effect in their robotics-based CT intervention (Z = 8.77, *p* < 0.01), which may reflect gender-based differences in technological affinity, interaction preferences, and task strategies.

Findings regarding educational level were mixed. Merino-Armero et al. (2022) reported a significant moderating effect of educational level on overall CT outcomes (Q = 4.560, *p* < 0.05), whereas Lai and Wong (2022) observed a significant effect only for affective competencies (Q = 11.96, *p* < 0.01), but not for cognitive competencies (Q = 2.06, *p* = 0.36), suggesting that its influence may depend on the targeted outcome dimension.

- Intervention design

Within the category of intervention design, two key moderating variables frequently examined are intervention duration and sample size. These variables, rooted in the implementation logic and allocation of instructional resources, play a crucial role in shaping both the effectiveness and stability of CT interventions. Multiple meta-analytic studies have demonstrated that these factors often function as significant or latent moderators across various educational contexts. Intervention duration is widely regarded as a core determinant of intervention outcomes. Sun et al. (2021) reported a significant moderating effect of duration on CT (Q = 14.522, *p* = 0.006). Similarly, Lai and Wong (2022), Zhang et al. (2024b), and Wang and Xie (2024) all included duration as a moderating variable in their analyses. Although not all studies reported statistically significant effects, intervention time was repeatedly highlighted as an explanatory factor for outcome variability, indicating its consistent potential as a moderator.

Sample size, as a variable related to statistical design, was also found to be a significant moderator in several studies. Sun et al. (2021) identified sample size as having a significant impact on intervention effectiveness (QB = 6.488, *p* = 0.009), and Sun et al. (2023) further confirmed the strength of this moderating effect in a meta-analysis of game-based CT interventions (Q = 22.022, *p* = 0.000).

- Instructional tools

Instructional tool variables encompass a range of intervention forms and platforms, including programming activity forms, programming environments, educational robots, game types, programming tools, and teaching styles. Numerous meta-analyses have explored the moderating effects of these variables, revealing substantial diversity and heterogeneity in outcomes. Regarding programming-related tools, Sun et al. (2021) found that programming activity forms did not significantly moderate the effectiveness of programming-based interventions (QB = 0.191, *p* = 0.662), suggesting that variations in activity design alone may not strongly influence learning outcomes. Similarly, Li et al. (2022) reported that interdisciplinary course contexts showed no significant moderating effect on unplugged activities (Q = 2.035, *p* > 0.05) or physical experiments (Q = 3.353, *p* > 0.05), indicating that cross-disciplinary settings may not systematically alter the impact of instructional tools.

In contrast, programming environments demonstrated partial moderating effects. Lai and Wong (2022) found a significant moderating role of environment type on cognitive competencies (Q = 19.98, *p* < 0.01), though not on affective competencies (Q = 2.71, *p* = 0.26), suggesting that environments have a stronger influence on skill acquisition than on motivation or attitude. However, Sun and Zhou (2023) observed no significant effect of text-based programming environments on intervention outcomes (Chi^2^ = 12.39, *p* = 0.088), indicating limited explanatory power in highly standardized instructional settings.

For game-based interventions, Lu et al. (2022) found that game type significantly moderated learning effectiveness (Q = 9.944, *p* = 0.041), highlighting possible cognitive mechanism differences among genres such as puzzle or strategy games. Conversely, Sun et al. (2023) reported no significant effect of game usage mode on the outcomes of educational games (Q = 5.382, *p* = 0.068), implying that the frequency or format of use alone may not predict learning gains.

In the domain of robotics and visualization tools, Hong (2024) found that teaching style significantly moderated the effectiveness of educational robots (Chi^2^ = 5.762, *p* = 0.020), emphasizing the importance of instructional pace and interaction patterns in technology-enhanced environments. Similarly, Xu et al. (2023) identified programming tools as a significant moderator (Z = 6.27, *p* < 0.001), suggesting that tool type and complexity influence cognitive load and strategy transfer.

Furthermore, Wang and Xie (2024) reported that scaffolding had a highly significant moderating effect on game-based learning (F = 46.076, *p* < 0.05), underscoring the value of instructional support systems in enhancing the impact of technology-driven interventions. Zhang et al. (2024b) reported a non-significant effect of intervention type on unplugged programming (Q = 2.664, *p* = 0.103), suggesting that this particular factor may not be a critical moderator in this context. Wang and Xie (2024) reported non-significant moderating effects of learning strategy on both CT concepts (Z = 1.371, *p* = 0.504) and CT practices (Z = 1.119, *p* = 0.572).

- Assessment methods

Assessment-related moderators include variables such as assessment types, assessment tools, and instrument formats. Existing studies suggest that the type and structure of assessments can significantly influence the measured effectiveness of CT interventions. For instance, Sun et al. (2021) found that assessment type significantly moderated the effects of programming-based interventions (Q = 5.317, *p* = 0.015), indicating that the use of tests, project-based assignments, or observational records may capture different dimensions of CT performance. Similarly, Wang and Xie (2024) found no significant moderating effects of assessment tools on either CT concepts (Z = 0.015, *p* = 0.992) or CT practices (Z = 0.021, *p* = 0.990), possibly due to the generalized nature of assessment dimensions or the limited structural validity of existing CT evaluation instruments.

Similarly, Sun and Zhou (2023) reported a significant moderating effect of assessment tools on the outcomes of text-based programming instruction (Chi^2^ = 7.46, *p* = 0.024), suggesting that the format and evaluative standards of the tool itself can shape how intervention effectiveness is estimated and interpreted. However, not all studies found significant effects. For example, Lu et al. (2022) reported that instrument type did not significantly moderate the outcomes of game-based learning interventions (Q = 0.496, *p* = 0.780), suggesting that in highly contextualized or immersive learning environments, variation in assessment format may not substantially alter learning processes or results.

### 3.3. Umbrella Review of Systematic Reviews

**Figure 7:**
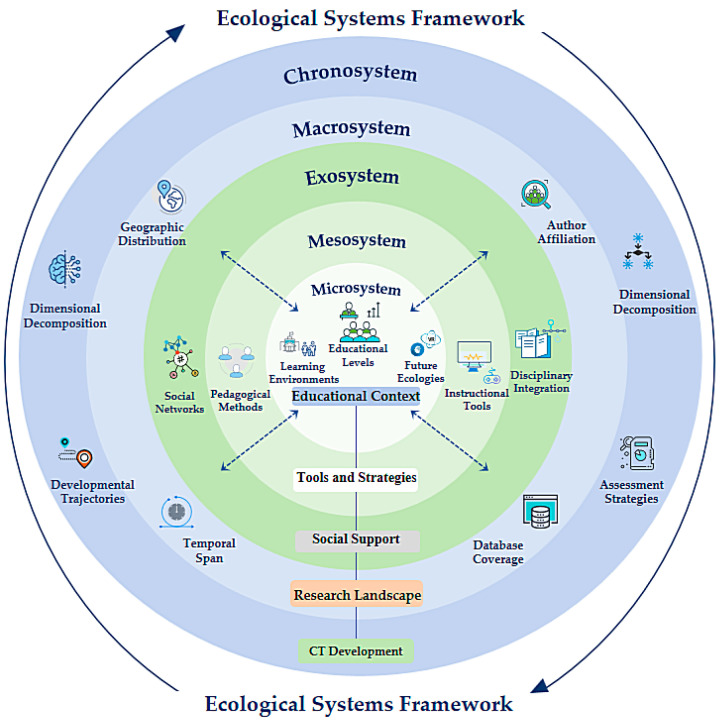
The ecological systems framework for analysing systematic reviews.

According to Bronfenbrenner (2000) ecological systems theory, the development of CT is influenced by complex interactions between environmental factors at multiple levels, providing an effective framework for understanding this process. For the 18 systematic reviews included in the study, we constructed a nested analytical framework comprising five hierarchical levels: the microsystem, the mesosystem, the exosystem, the macrosystem and the chronosystem (Bronfenbrenner 1979). This framework encompasses dimensions such as skill development, macro-level characteristics, social support, tools and strategies, educational environments and processes (see Figure 7). These dimensions reflect varied research orientations and methodological approaches. The five elements in the framework—visualized in the ecological systems framework (see Figure 6)—are not mutually exclusive, but rather derived from a synthesis of thematic focus and analytical emphasis across studies.

#### 3.3.1. Microsystem

The microsystem, representing the immediate educational context, refers to the direct learning environments such as classrooms, households, and informal learning settings. Among the included reviews, this dimension is the most frequently addressed, with a strong emphasis on how CT instruction is contextually adapted across direct learning environments, educational levels, and subject domains.

The influence of family factors within the educational microsystem is a significant area of inquiry. For example, Cai and Wong (2024) established a contextualized theoretical framework titled “Parental Involvement in CT Education.” Their systematic review revealed the deep influence of parental engagement by analyzing its affective, behavioral, and cognitive outcomes on children’s CT learning processes. Beyond the home, studies also zeroed in on the adaptability of CT assessment within diverse educational settings. Tang et al. (2020) examined CT assessment implementation across various contexts by identifying four analytical dimensions: educational context, assessment construct, assessment type, and reliability/validity evidence. Their work proposed a dual-focus framework that integrates contextual adaptation with the structural analysis of assessment tools, advocating for a teaching-assessment integration approach.

Providing a comprehensive view, Lee et al. (2022) analyzed implementation across educational levels (elementary to high school), instructional design, pedagogical strategies, and the use of specific technologies like robotics, block/text-based programming, and unplugged activities. Reinforcing context-dependent patterns, Hsu et al. (2018) systematically detailed the relationships among learners, curricular subjects, and instructional strategies, identifying age-related patterns in pedagogical approaches and participation. Furthermore, Bati (2022) focused on CT skill development in children aged 3–6 years across plugged-in and unplugged contexts, emphasizing developmental stage, gender, and cognitive load as critical design factors and revealing issues in aligning age with learning media.

In addition to synthesizing existing findings, some reviews also engaged in theoretical construction. Tikva and Tambouris (2021b) developed the CTPK-12 model through systematic review, integrating contextual variables such as learning strategies, tools, assessment, capacity building, and learner factors. This model provides a comprehensive portrayal of CT development processes in K–12 programming education. Lai et al. (2023) specifically analyzed how contextual variables—such as partnership, learner roles, peer interaction, classroom culture, task nature, and scaffolding—moderate the effectiveness of collaborative learning. This work exemplifies process-oriented contextual research by exploring how collaborative strategies are adapted across educational stages and learning environments.

#### 3.3.2. Mesosystem

The mesosystem, conceptualized here as the tools and strategies layer, functions as a bridge connecting various educational contexts within the microsystem. This category constitutes the second most represented focus in the reviewed literature, after studies on educational context. Research in this area centers on how instructional tools, pedagogical methods, and their combinatory designs influence the development of CT. These studies explore not only the types of technological media—such as programming platforms, language tools, and interactive devices—but also how instructional strategies (e.g., collaborative, game-based, project-based learning) are paired with tools to shape learning pathways and regulate cognitive load.

For example, Zhang and Nouri (2019) synthesized evidence on how the Scratch programming tool supports CT development among K–9 students. Expanding beyond single-tool analyses, Jin and Cutumisu (2024) examined how teaching methods, technological tools, and assessment approaches interact during CT instruction, particularly focusing on transfers from CT to deeper learning in K–12 contexts. Some studies have emphasized the interplay between media, tools, and strategies. Hsu et al. (2018), for instance, conducted a broad synthesis that highlighted the synergistic role of various instructional media and pedagogical techniques. Their review examined tools such as Scratch, Alice, and LEGO, and categorized strategies like project-based learning, collaborative learning, game-based learning, and unplugged activities. Adding an assessment perspective, Rao and Bhagat (2024) analyzed the combined use of instructional tools, pedagogical strategies, and assessment practices, emphasizing their functional roles and contextual applications in CT instruction.

Fagerlund et al. (2021) focused specifically on Scratch programming, identifying effective content and activity strategies that promote CT development and discussing how these can be operationalized in instruction and formative assessment, underscoring the pivotal role of tool–strategy integration. In the domain of game-based learning (GBL), Wang et al. (2023) reviewed a wide range of tools and strategies employed in CT education, incorporating game mechanics such as feedback and challenge to construct a constructivist-aligned instructional framework. Similarly, Tikva and Tambouris (2021a) integrated tool and strategy elements within their CTPK-12 model, mapping their embedded roles in competency development, curriculum design, and teacher capacity building, thereby illustrating a theoretically grounded tool–strategy–system integration pathway.

#### 3.3.3. Exosystem

The exosystem represents the broader social context that indirectly influences learners, with a particular focus on social support networks and interdisciplinary connections. This layer reflects the expanding paradigm of CT education, emphasizing how learners’ social networks, family backgrounds, and disciplinary integration shape the development of CT competencies. For instance, Yeni et al. (2024) conducted a systematic review on interdisciplinary CT instruction in K–12 settings. The study examined instructional strategies, technologies and tools, and assessment practices, revealing that most interdisciplinary CT teaching remains at the substitution level rather than achieving transformative integration. This suggests that while cross-disciplinary efforts are increasing, deep pedagogical integration is still limited. Focusing on specific subject areas, Ye et al. (2023) reviewed the integration of CT in K–12 mathematics education, identifying that student-centered instruction and geometrized programming effectively support the co-development of CT and mathematical reasoning. The study emphasized that CT-driven math learning involves interactive and iterative processes that align mathematical thinking with computational logic. The study proposed targeted strategies around scaffolding and collaborative instructional design, highlighting how social dynamics interact with pedagogical frameworks. Collectively, these studies underscore that CT education is a product of dynamic interactions within the broader social system. Its effectiveness is not solely determined by instructional content or design, but also shaped by familial, cultural, and technological environments.

#### 3.3.4. Macrosystem

The macrosystem focuses on the synthesis of macro-level characteristics, offering a broader landscape of CT research distribution and its developmental trajectory. This layer typically uses variables such as country of origin, author affiliation, study quantity, and publication distribution to portray the structural evolution of the CT research field. Among the 18 reviewed systematic literature reviews, high-frequency macro-level variables included sample characteristics, geographic distribution, subject domain, temporal span, and database coverage.

For instance, Zhang and Nouri (2019) systematically analyzed databases, publication years, countries, sample sizes, subject areas, and Scratch artefacts, constructing a structural ecological map of CT research. Lee et al. (2022) and Cai and Wong (2024) incorporated at least four macro-level dimensions in their analyses, integrating these into broader discussions on contextual environments and social mechanisms. Their work highlights the interplay between data characteristics and theoretical construction. Taking geographic distribution as an example, Hsu et al. (2018) reviewed 120 studies and identified significant regional disparities in CT research output, with a high concentration in North America and East Asia. Similarly, Lee et al. (2022) treated country/region as a contextual factor, noting its indirect impact on educational adaptability and relevance.

In terms of temporal span, Hsu et al. traced the growth trajectory of CT research between 2006 and 2017, while Cai and Wong (2024) extended the analysis over a ten-year period to examine the shifting research focus on parental involvement in CT education. Regarding sample characteristics, Cai and Wong (2024) provided a detailed breakdown of age groups, sample sources, and size variations, assessing how these differences influenced the generalizability of research findings.

On the research methods dimension, Hsu et al. (2018) categorized the reviewed literature into quantitative, qualitative, and mixed-methods, offering insights into the methodological evolution of the field. Zhang and Nouri (2019) also analyzed disciplinary scope and database coverage, revealing how CT research is embedded in computer science, mathematics, and interdisciplinary domains, and conducted cross-validation across Web of Science and Scopus.

#### 3.3.5. Chronosystem

The chronosystem emphasizes the temporal dynamics of learners’ development, centering on the progressive evolution of CT competencies over time. Research on the chronosystem has primarily yielded two key conclusions: a detailed mapping of skill acquisition and the identification of a significant conceptual gap.

The primary focus has been on decomposing and mapping the developmental trajectories of CT concepts and practices. Studies have operationalized the stepwise acquisition of these skills across different age groups and educational levels. A representative study is Zhang and Nouri (2019), who adopted Brennan and Resnick (2012) three-dimensional model—concepts, practices, and perspectives—to analyze CT across age groups. Their work exemplifies how Scratch supports the stepwise acquisition of CT in early programming education, offering a micro-level view of skill development. Similarly, Fagerlund et al. (2021) built upon existing understandings of core CT by proposing a set of Computational Thinking Core Educational Principles (CEPs). These principles were operationalized into a structured CT competency scale tailored for primary education, addressing learning, instruction, and assessment needs in early schooling. Moreover, analyses concentrating on CT concepts and practices have revealed that factors such as instructional design, learning tools, and AI interactions contribute to the refinement and layering of students’ CT competencies within authentic learning contexts (Weng et al. 2024). This contextual analysis extends to game-based learning, where specific game mechanics have been mapped to impacts on CT sub-skills, providing one of the few intricate dissections of skill development within gamified environments (Wang et al. 2023).

Despite these advances in mapping concepts and practices, a significant gap exists regarding the perspectives dimension of CT, encompassing its social, reflective, and metacognitive aspects. Underrepresentation of Perspectives: Most existing studies disproportionately focus on concepts and practices, suggesting a narrowed understanding of CT that overlooks its broader cognitive and societal functions. To address this, a few models have been proposed to expand the scope of CT to a multidimensional foundation that integrates concepts, skills, practices, perspectives, and attitudes (Tikva and Tambouris 2021b). The field faces several persistent limitations that hinder the development of a truly temporally sensitive framework. These include a lack of longitudinal studies, insufficient integration of advanced cognitive network analyses, and the use of non-standardized assessment instruments, especially for the nuanced measurement of the underrepresented perspectives dimension. Addressing these deficiencies is essential for constructing a comprehensive framework of CT progression.

## 4. Discussion and Limitations

This umbrella review synthesizes evidence on CT education by analyzing 33 systematic reviews and meta-analyses, which focus on intervention effects, methodological trends, and contextual moderators. The findings highlight the multifaceted nature of CT pedagogy, identifying project-based learning, text-based programming, and game-based learning as consistently high-impact approaches. We identified four key evidence-based moderators—learner characteristics, intervention design, instructional tools, and assessment methods—that contribute to the advancement of CT education. This review also discusses existing knowledge gaps and offers recommendations for future research and practice.

### 4.1. What Is the Quality of Meta-Analyses and Systematic Reviews Related to CT, and What Overall Trends Do They Reflect?

The AMSTAR 2 assessment revealed distinct quality profiles between evidence syntheses on CT. Meta-analyses demonstrated greater methodological rigor, with most achieving moderate to high-quality ratings by adhering to core procedures like systematic literature screening and data extraction. There was very high heterogeneity (I^2^ = 82.4%) when all studies were included. After excluding extreme effect values, regression analysis of grouped elements indicates that sample quality is a factor contributing to this heterogeneity. A limitation of this study is that this relationship is significant only when extreme effect values are excluded; nevertheless, it reveals that research quality may be a weaker but nonetheless present moderating factor for effect sizes.

Conversely, systematic reviews exhibited lower overall quality, with a majority rated as moderate to low and none reaching the high-quality threshold. This disparity indicates that while meta-analyses offer superior procedural standardization, data synthesis, and bias control, systematic reviews suffer from significant limitations, especially in research framework development, process transparency, and bias mitigation. Although systematic reviews hold substantial potential as tools for evidence synthesis, their current limitations demand methodological refinement. To ensure evidence synthesis reliably guides educational technology practice, future studies must prioritize quality enhancement: all reviews should be encouraged to register their protocols, employ dual independent procedures for critical steps, and strengthen the disclosure of conflicts of interest. These improvements are critical for enhancing the credibility and practical utility of the entire CT evidence base.

The current meta-analysis research in CT shows an evolutionary trend of “centralized initiation, rapid development, and gradual updates,” whereas systematic literature reviews follow a pattern of “sporadic exploration, rapid accumulation, and widespread dissemination.” The development of these research trends can be attributed to several factors. First, many countries worldwide have implemented policies incorporating CT into basic education curricula (Izquierdo-Álvarez and Pinto-Llorente 2025). For example, the U.S. Computer Science Education for All (CSE4All) program and similar digital competency initiatives in Europe and Asia emphasize developing essential digital-age skills, with CT as a key component (Li et al. 2020; Menolli and Neto 2022). These policy directions have facilitated meta-analysis research by promoting the evaluation of methods and effectiveness in CT education. Second, the COVID-19 pandemic has accelerated the development of digital learning environments, increasing the demand for CT interventions. In response, educators and researchers must quickly assess the effectiveness of various online CT approaches to inform policy development (Guo et al. 2024). This has led to the rise of short-term, focused meta-analytic studies aimed at swiftly synthesizing relevant evidence. Finally, educators must understand which instructional strategies and tools are most effective for developing students’ CT. Meta-analysis offers a systematic approach to synthesizing findings across studies, aiding educators in making evidence-based decisions (Merino-Armero et al. 2022). This practical need has driven the growth of meta-analytic studies, enabling more accurate assessments of the effectiveness of various teaching methods.

### 4.2. How Effective Are Different Types of CT Intervention Strategies, and What Key Moderating Variables Influence Their Outcomes?

This study systematically reviews existing evidence to emphasise the importance of explicit and effective intervention strategies in developing CT and addressing complex societal challenges. Of the various instructional models examined, project-based learning, text-based programming and robot-based programming were found to be particularly effective. This student-centered model promotes deep learning and stimulates motivation as students actively construct knowledge through inquiry and practice (Pastor et al. 2025). Concurrently, this research has also revealed that traditional curriculum-based instruction, with its structured teaching design and systematic knowledge transfer, can similarly yield above-average intervention effects, providing a stable pathway for teaching foundational CT concepts (Paleenud et al. 2024). Furthermore, experiential learning—particularly approaches leveraging physical robots—creates embodied computational environments through direct hardware interaction and immediate feedback, effectively facilitating the practical internalization of CT (Valls Pou et al. 2022). Collectively, these findings reveal that diverse intervention strategies can complement each other in CT development, providing a foundation for systematic curriculum design and pedagogical practice.

In terms of programming formats, text-based programming, robotics programming and computer-free programming all have an influence on outcomes in CT, but the effectiveness of each varies. Text-based programming requires learners to be precise with syntax and logic, which helps develop abstract thinking, algorithmic thinking, and debugging skills (Sun and Zhou 2023). The results of this review also suggest that robot programming is better suited for beginners and perception-driven tasks. Unlike text-based programming, robot programming often uses a graphical interface with lower cognitive demands, making it ideal for beginners. However, the simplicity of robot programming makes it challenging for students to progress deeply without systematic programming training (Sun et al. 2024). Finally, the effectiveness of computerless programming largely depends on task design. If the task is cognitively challenging and logically structured, it can effectively promote the development of CT (Fanchamps et al. 2024; Threekunprapa and Yasri 2021). Conversely, if the task is too simple, it will fail to achieve the desired effect.

This review analyzes the characteristics and effects of individual, collaborative, and gamified learning modalities, offering educators valuable guidance for selecting and combining these methods in various instructional contexts. Individual learning, particularly solo problem-solving, may be limited by factors such as insufficient cognitive activation, limited strategy transfer, low motivation, and lack of social interaction in enhancing CT (Hsu and Chen 2025). Gamified learning emerged as the most prominent approach in this study. Gamified learning incorporates game elements like points, rewards, and challenges, significantly boosting learners’ motivation and engagement. Additionally, gamified learning typically involves lower risk and stress, helping to reduce learners’ anxiety. Learning in a relaxed and enjoyable environment enhances learning outcomes (Kuo and Hou 2025). Collaborative learning is also an important learning style. Collaborative learning fosters interaction and communication through group discussions and cooperative problem-solving. These interactions stimulate cognitive conflicts, prompting students to think more deeply about problems (Kio 2016).

In terms of moderating variables, this study identifies four key factors that collectively moderate the ultimate outcomes of educational interventions: learner characteristics, intervention design, instructional tools and assessment methods. Learner characteristics moderately influence CT outcomes by affecting receptivity and limiting intervention effectiveness (Wongwatkit et al. 2020). Specifically, gender and subject background show stable, interpretable effects, while the influence of grade level and educational stage is conditional and context-sensitive, requiring further interpretation alongside intervention design and assessment. Future research should use hierarchical or interaction models to better delineate the boundary conditions and moderating pathways of these characteristics across CT strategies.

Meanwhile, intervention design factors, like duration and sample size, determine intervention intensity and generalisability, thereby affecting outcome stability and replicability. Smaller-sample studies may yield larger effect sizes due to tighter control, but their generalizability and external validity require cautious interpretation.

Instructional tools, as the medium for delivery, must be aligned with students’ learning styles and cognitive needs in terms of both type and functional suitability to ensure intervention feasibility and engagement. Their consistent significance in specific contexts emphasizes that tool selection must align critically with the task type and learning objectives. Future research should explore the interaction among tool characteristics, task complexity, and learner cognitive styles to clarify moderating mechanisms along the tool–process–outcome pathway.

Finally, assessment methods clearly moderate outcomes, especially when assessment type aligns highly with instructional objectives, enhancing the accuracy of intervention effect detection through increased sensitivity. Conversely, lack of standardized tools or vague indicators often prevents moderating effects from reaching significance, requiring urgent refinement. Future research should prioritize refining assessment classification, diversifying CT tools, and investing in validity testing to improve both interpretive precision and practical utility.

### 4.3. How Are Factors Influencing CT Development Distributed Across System Ecological Levels (Individual, Micro, Meso, Exo, Macro)?

This review systematically organizes 18 systematic review articles, collectively constructing a multidimensional knowledge structure for CT education research around five complementary elements: macro-level characteristics, contextual processes, CT development, tools and strategies, and social support and interdisciplinary extension. Despite variations in methodology, an overarching trend of interdisciplinary convergence emerges, reflecting the field’s evolutionary trajectory toward integrated modeling. The macro-level (quantitative mapping) serves as a critical foundation for systematic reviews, providing background to contextualize the development and subsequent interpretation of findings; the contextual level emphasizes adaptation pathways across educational stages; the skill-based level refines the structure of CT competencies; the tool-based level stresses intervention mechanisms and technology-mediated strategies; and the social-based level integrates social support and interdisciplinary extension, reinforcing the socio-ecological positioning of CT education. Overall, these elements form a complementary framework in terms of analytical units, theoretical pathways, and explanatory scope.

However, the current body of research faces significant limitations that impede the construction of comprehensive theoretical frameworks. Most studies still focus on conventional learning contexts (e.g., classrooms, homes) with insufficient attention to emerging environments like VR-based instruction and AI-assisted learning, which limits current models’ adaptability to future learning ecologies. Furthermore, a key constraint lies in content homogeneity, as many studies rely on a narrow set of commonly used tools and pedagogical strategies (e.g., Scratch, project-based learning), resulting in substantial methodological overlap that limits the diversity, generalizability, and novelty of conclusions. Crucially, the absence of longitudinal evidence restricts deeper insight into the mechanisms of CT learning across time, and current research suffers from insufficient integration of cognitive network analyses and non-standardized assessment instruments for nuanced skill measurement. To advance CT education studies from fragmented descriptions toward systematic modeling, future research must expand the empirical base, address structural inequalities that influence access, and maintain an analytical focus while promoting the multidimensional integration of these five elements. Adopting a social-ecological modeling approach is urgently needed to capture the complexity of these influences, construct more explanatory theoretical frameworks, and provide strategic guidance for inclusive and temporally sensitive CT educational practice.

### 4.4. Limitations

This umbrella review systematically synthesizes existing evidence in CT education; however, it’s important to acknowledge several key limitations. Although this study prioritized meta-analyses containing the most comprehensive or recent evidence in its inclusion criteria to avoid content duplication, overlap among original studies remains an inherent limitation of umbrella reviews. These limitations do not invalidate the patterns we identified, but caution is warranted when generalizing the findings. Future research may employ overlapping rate quantification techniques to more precisely assess and mitigate such biases. Furthermore, while this umbrella review systematically organized evidence across levels based on ecosystem theory, the full complexity of ecosystems remains challenging to capture comprehensively within a review. For instance, the theory itself implies intricate dynamic interactions between systems, yet existing empirical studies offer limited direct exploration of cross-level effects, presenting a significant avenue for future research.

## 5. Conclusions and Future Directions

The importance of CT as a core competency is increasingly evident. This umbrella review provides a comprehensive synthesis of 33 systematic reviews and meta-analyses in the field of CT, summarising evidence from multiple studies. A systematic evaluation of the methodological quality of the included literature is conducted, and overall research trends in CT are identified. Furthermore, by synthesising meta-analyses, the study identifies clusters of intervention strategies, such as instructional models, programming formats and learning modalities, and validates the effectiveness of different approaches. Four key moderating variables are identified that constitute effective intervention pathways: learner characteristics, intervention design, instructional tools, and assessment methods. Additionally, this research integrates CT development studies from an ecosystem theory perspective, mapping components from micro to macro levels. This review provides teachers, researchers and policymakers with significant theoretical and practical reference value, enabling them to take effective action to foster CT based on specific contexts and needs. Future research should capture the developmental mechanisms of CT in real-world settings, analyse issues related to implementation conditions and develop tailored, differentiated interventions for CT. There should also be an emphasis on developing standardised diagnostic tools for the precise assessment and identification of CT outcomes. This will establish an initial evidence base for CT education and provide insight into learners’ cognitive development.

## Data Availability

The original contributions presented in this study are included in the article. Further inquiries can be directed to the corresponding author.

## Appendix A

**Table A1:** Quality assessment table of meta-analyses.

ID	Authors (Year)	A1	A2	A3	A4	A5	A6	A7	A8	A9	A10	A11	A12	A13	A14	A15	A16	Total
		Yes (Y), No (N)	Yes (Y), Partial Yes (PY), No (N)	Y, N	Y, PY, N	Y, N	Y, N	Y, PY, N	Y, PY, N	Y, PY, N	Y, N	Y, N	Y, N	Y, N	Y, N	Y, N	Y, N	0–16
1	Fidai et al. (2020)	Y	PY	Y	Y	N	N	N	Y	Y	N	Y	Y	Y	Y	Y	Y	11.5
2	Sun et al. (2021)	Y	PY	Y	Y	Y	Y	N	Y	Y	N	Y	Y	Y	Y	Y	Y	13.5
3	Zhang et al. (2021)	Y	N	Y	Y	Y	Y	N	Y	Y	N	Y	Y	Y	Y	Y	Y	13
4	Merino-Armero et al. (2022)	Y	Y	Y	Y	Y	Y	N	Y	PY	N	Y	N	N	Y	Y	Y	11.5
5	Li et al. (2022)	Y	PY	Y	Y	Y	Y	PY	Y	N	Y	N	N	N	Y	Y	N	10
6	Lai and Wong (2022)	Y	N	Y	Y	Y	Y	Y	Y	N	N	Y	N	N	Y	Y	Y	11
7	Sun and Zhou (2023)	Y	N	Y	Y	Y	Y	N	Y	N	N	Y	N	N	Y	Y	Y	10
8	Lu et al. (2022)	Y	N	Y	PY	Y	Y	N	Y	N	N	Y	N	N	Y	Y	Y	10.5
9	Hong (2024)	Y	N	Y	Y	PY	Y	Y	N	N	N	Y	N	N	Y	Y	Y	10
10	Sun et al. (2023)	Y	N	Y	Y	Y	Y	N	Y	N	N	Y	N	N	Y	Y	Y	11
11	Xu et al. (2023)	Y	N	Y	Y	Y	Y	N	Y	N	N	Y	N	N	Y	Y	Y	11
12	Wang et al. (2024)	Y	N	N	Y	Y	Y	Y	N	PY	N	N	N	N	Y	Y	Y	10
13	Zhang et al. (2024b)	N	Y	Y	PY	Y	Y	N	PY	N	N	Y	N	N	Y	Y	Y	10
14	Wang and Xie (2024)	Y	N	Y	PY	Y	Y	N	Y	N	N	Y	N	N	Y	Y	Y	10.5
15	Zhang et al. (2024a)	Y	N	Y	Y	Y	Y	N	Y	N	N	Y	N	N	Y	Y	Y	10

**Table A2:** Quality assessment table of systematic reviews.

ID	Authors (Year)	A1	A2	A3	A4	A5	A6	A7	A8	A9	A10	A16	Total
		Yes (Y), No (N)	Yes (Y), Partial Yes (PY), No (N)	Y, N	Y, PY, N	Y, N	Y, N	Y, PY, N	Y, PY, N	Y, PY, N	Y, N	Y, N	0–11
1	Hsu et al. (2018)	N	N	Y	Y	N	Y	N	Y	N	N	N	4
2	Zhang and Nouri (2019)	N	N	Y	Y	Y	Y	N	Y	N	N	N	5
3	Fagerlund et al. (2021)	N	N	Y	Y	N	N	N	Y	N	N	Y	4
4	Tang et al. (2020)	N	N	Y	Y	Y	Y	N	Y	N	N	Y	6
5	Tikva and Tambouris (2021a)	N	N	Y	Y	Y	N	Y	Y	N	N	Y	6
6	Tikva and Tambouris (2021b)	N	N	Y	Y	Y	Y	N	Y	N	N	Y	6
7	Bati (2022)	N	N	Y	Y	N	N	N	Y	Y	N	Y	5
8	Lee et al. (2022)	N	N	Y	Y	Y	Y	Y	N	N	N	Y	6
9	Lai et al. (2023)	N	N	Y	Y	Y	Y	N	Y	N	N	Y	6
10	Cai and Wong (2024)	N	N	Y	Y	Y	Y	N	Y	N	N	Y	6
11	Ye et al. (2023)	N	N	Y	N	Y	Y	N	PY	N	N	Y	4.5
12	Wang et al. (2023)	N	N	Y	Y	Y	Y	N	PY	N	N	Y	5.5
13	Yin et al. (2024)	N	N	Y	Y	Y	Y	N	Y	Y	N	N	6
14	Yeni et al. (2024)	N	N	Y	Y	Y	Y	N	Y	N	N	Y	6
15	Weng et al. (2024)	N	N	Y	Y	Y	Y	N	Y	N	N	Y	6
16	Rao and Bhagat (2024)	N	N	Y	Y	N	N	Y	Y	N	N	Y	5
17	Espinal et al. (2024)	N	N	Y	Y	N	N	N	Y	N	N	Y	4
18	Jin and Cutumisu (2024)	Y	N	Y	Y	Y	N	Y	N	N	N	Y	6

**Table A3:** Coding table for meta-analysis studies.

ID	Authors (Year)	Database	Time Span	Number of Studies	Number of Effect Sizes	Total Sample Size	Moderator Variables	Independent Variables	Outcome Variables	Effect Size Type	Effect Size	Confidence Interval	*p* Value	Q (P-Value)	I-squared	Publication Bias
1	Fidai et al. (2020)	ERIC, PsyCINFO, Web of Science, LearnTechLib	2010–2019	12	29	584	grade level, study duration	Arduino-based interventions	CT skills	Cohen’s d	1.03	[0.630, 1.420]	<0.001	40.85 (0.000)	87.32%	Y
2	Sun et al. (2021)	ScienceDirect, Spring, Web of Science	1984–2021	54	114	11,827	Subject, Sample size, Intervention duration, Programming activity forms, Programming instruments, Assessment types	Solo programming	CT skills	Hedges’ g	0.622	[0.442, 0.801]	<0.001	854.321 (0.000)	86.77%	Y
				60				Collaborative programming			0.670	[0.060, 0.552]	<0.001			Y
3	Zhang et al. (2021)	Web of Science, ERIC, IEEE, ScienceDirect, Springer Link	2010–2019	6	6	930	Gender, Grade Levels, Experimental Periods	Educational Robots	CT skills	SMD	0.48	[0.32, 0.64]	<0.001	NA	86.00%	Y
4	Merino-Armero et al. (2022)	Web of Science Core Collection, ProQuest, ERIC, PubMed, EBSCO, etc.	before 2020	41	61	3852	Educational level, Educational area, Kind of intervention, Type of learning tool, Assessment tool, Framework used, Session length, Intervention length, Intervention intensity, CT dimension worked	CT education	CT skills	Cohen’s d	1.044	[0.849, 1.238]	<0.001	375.5 (0.000)	86%	Y
5	Li et al. (2022)	Web of Science, EBSCO, Taylor & Francis, ScienceDirect, Springer	2006–2021	29	31	2764	Grade level, Interdisciplinary course, Experiment duration	Unplugged activities	CT skills	Hedges’ g	0.392	[0.308, 0.475]	<0.001	86.138 (0.000)	83.75%	Y
								Programming exercises			0.576	[0.408, 0.734]	<0.001			Y
6	Lai and Wong (2022)	ACM Digital Library, IEEE Xplore, ERIC, Scopus	2000–2021	33	220	4717	Educational level, Programming environment, Duration of study, Grouping method, Group size, Educational level	Collaborative problem solving	CT skills	Hedges’ g	0.562	[0.08–1.04]	<0.001	NA	NA	NA
								Individual problem solving			0.316	[0.10–0.53]	<0.001	NA	NA	NA
7	Sun and Zhou (2023)	ScienceDirect, Spring and Web of Science	2006–2022	19	37	NA	Education level, Intervention duration, Text-based Programming Environment, Assessment tools, Sample size	Text-based programming	CT skills	Hedges’ g	0.71	[0.51, 0.90]	<0.001	176.05 (0.000)	82.22%	Y
8	Lu et al. (2022)	EBSCO, Web of Science, ProQuest, ScienceDirect, CNKI, WanFang DATA	2011–2022	24	28	2134	Game type, Intervention duration, Grade level, Instrument type	Game-based learning	CT skills	Hedges’ g	0.677	[0.532, 0.821]	<0.001	117.264 (0.000)	76.98%	Y
9	Hong (2024)	Web of Science, ERIC, SpringerLink, EBSCOhost, IEEE, ScienceDirect, Google Scholar	2010–2023	27	36	NA	Grade Levels, Teaching styles, Participation methods, Experimental cycles, Sample size	Educational robots	CT skills	SMD	0.558	[0.419,0.697]	<0.001	149.608 (0.000)	76.61%	Y
10	Xu et al. (2023)	Web of Science, ScienceDirect, Google Scholar	2010–2020	22	39	NA	Sample size, Grade level, Game usage mode, Game tool	Educational games	CT skills	Hedges’ g	0.766	[0.580, 0.951]	<0.001	311.834 (0.000)	87.81%	Y
11	Xu et al. (2023)	Web of Science Core, ERIC, ScienceDirect	2000–2021	28	98	4154	Learning stage, Intervention duration, Learning scaffold, Programming tool, Evaluation tool	Programming teaching	CT skills	SMD	0.72	[0.60, 0.83]	<0.001	NA	88%	Y
12	Wang et al. (2024)	Web of Science	2019–2023	17	35	1665	Gender, Education level, Scaffolding, Intervention Length	Empirical interventions	CT skills	Cohen’s d	0.83	[0.730, 0.890]	<0.001	249.236 (0.000)	88.26%	Y
13	Zhang et al. (2024b)	Web of Science, ERIC, IEEE, ScienceDirect, Springer Link, Google Scholar	2006-2023	15	22	NA	School level, Gender, Study duration, Subject, UP categories	Unplugged programming activities	CT skills	Hedges’ g	0.631	[0.463, 0.799]	<0.001	NA	75%	Y
14	Wang and Xie (2024)	Web of Science, Google Scholar, Science Direct	2012–2022	26	33	3381	Grade Level, Study duration, Culture, Learning strategy, Assessment tools	robot-supported learning	CT skills	Hedges’ g	0.643	[0.528, 0.757]	<0.001	105.082 (0.000)	69.45%	Y
15	Zhang et al. (2024a)	IEEE Xplore, ScienceDirect, Web of Science, CNKI	2013–2023	31	NA	NA	Educational stages	project-based learning	CT skills	SMD	0.57	[0.50, 0.66]	<0.001	577.66 (0.000)	78%	Y

## References

[B1-jintelligence-13-00157] Alene Kefyalew Addis, Hertzog Lucas, Gilmour Beth, Clements Archie CA, Murray Megan B. (2024). Interventions to prevent post-tuberculosis sequelae: A systematic review and meta-analysis. EClinicalMedicine.

[B2-jintelligence-13-00157] Ballard Evan David, Haroldson Rachelle (2021). Analysis of Computational Thinking in Children’s Literature for K-6 Students: Literature as a Non-Programming Unplugged Resource. Journal of Educational Computing Research.

[B3-jintelligence-13-00157] Bati Kaan (2022). A systematic literature review regarding computational thinking and programming in early childhood education. Education and Information Technologies.

[B4-jintelligence-13-00157] Bers Marina Umaschi, Strawhacker Amanda, Sullivan Amanda (2022). The state of the field of computational thinking in early childhood education. OECD Education Working Papers.

[B5-jintelligence-13-00157] Bocconi Stefania (2016). Developing Computational Thinking in Compulsory Education.

[B6-jintelligence-13-00157] Borenstein Michael, Hedges Larry V., Higgins Julian P. T., Rothstein Hannah R. (2009). Effect Sizes Based on Means. Introduction to Meta-Analysis.

[B7-jintelligence-13-00157] Brennan Karen, Resnick Mitchel (2012). New frameworks for studying and assessing the development of computational thinking. Paper presented at 2012 Annual Meeting of the American Educational Research Association.

[B8-jintelligence-13-00157] Bronfenbrenner Urie (1979). The Ecology of Human Development.

[B9-jintelligence-13-00157] Bronfenbrenner Urie (2000). Ecological Systems Theory.

[B10-jintelligence-13-00157] Cai Haiyan, Wong Gary K. W. (2024). A systematic review of studies of parental involvement in computational thinking education. Interactive Learning Environments.

[B11-jintelligence-13-00157] Camargo Salamanca Sandra Liliana, Parra-Martínez Andy, Chang Ammi, Maeda Yukiko, Traynor Anne (2024). The Effect of Scoring Rubrics Use on Self-Efficacy and Self-Regulation. Educational Psychology Review.

[B12-jintelligence-13-00157] Espinal Alejandro, Vieira Camilo, Magana Alejandra J. (2024). Professional Development in Computational Thinking: A Systematic Literature Review. ACM Transactions on Computing Education.

[B13-jintelligence-13-00157] Fagerlund Janne, Häkkinen Päivi, Vesisenaho Mikko, Viiri Jouni (2021). Computational thinking in programming with Scratch in primary schools: A systematic review. Computer Applications in Engineering Education.

[B14-jintelligence-13-00157] Fanchamps Nardie, van Gool Emily, Slangen Lou, Hennissen Paul (2024). The effect on computational thinking and identified learning aspects: Comparing unplugged smartGames with SRA-Programming with tangible or On-screen output. Education and Information Technologies.

[B15-jintelligence-13-00157] Fidai Aamir, Capraro Mary Margaret, Capraro Robert M. (2020). “Scratch”-ing computational thinking with Arduino: A meta-analysis. Thinking Skills and Creativity.

[B16-jintelligence-13-00157] Guo Shuchen, Zheng Yuanyuan, Zhai Xiaoming (2024). Artificial intelligence in education research during 2013–2023: A review based on bibliometric analysis. Education and Information Technologies.

[B17-jintelligence-13-00157] Gutiérrez-Núñez Sandra Erika, Cordero-Hidalgo Aixchel, Tarango Javier (2022). Implications of Computational Thinking Knowledge Transfer for Developing Educational Interventions. Contemporary Educational Technology.

[B18-jintelligence-13-00157] Hong Lan (2024). The impact of educational robots on students’ computational thinking: A meta-analysis of K-12. Education and Information Technologies.

[B19-jintelligence-13-00157] Hsu Ting-Chia, Chen Mu-Sheng (2025). Effects of students using different learning approaches for learning computational thinking and AI applications. Education and Information Technologies.

[B20-jintelligence-13-00157] Hsu Ting-Chia, Chang Shao-Chen, Hung Yu-Ting (2018). How to learn and how to teach computational thinking: Suggestions based on a review of the literature. Computers & Education.

[B21-jintelligence-13-00157] Izquierdo-Álvarez Vanessa, Pinto-Llorente Ana María (2025). Exploring Pre-Service Teachers’ Perceptions of the Educational Value and Benefits of Computational Thinking and Programming. Sustainability.

[B22-jintelligence-13-00157] Jin Hao-Yue, Cutumisu Maria (2024). Cognitive, interpersonal, and intrapersonal deeper learning domains: A systematic review of computational thinking. Education and Information Technologies.

[B23-jintelligence-13-00157] Kafai Yasmin B., Sawyer R. K. (2005). Constructionism. The Cambridge Handbook of the Learning Sciences.

[B24-jintelligence-13-00157] Kanaki Kalliopi, Chatzakis Stergios, Kalogiannakis Michail (2025). Fostering Algorithmic Thinking and Environmental Awareness via Bee-Bot Activities in Early Childhood Education. Sustainability.

[B25-jintelligence-13-00157] Kio Su Iong (2016). Extending social networking into the secondary education sector. British Journal of Educational Technology.

[B26-jintelligence-13-00157] Kite Vance, Park Soonhye, Wiebe Eric (2019). Recognizing and Questioning the CT Education Paradigm. Paper presented at 50th ACM Technical Symposium on Computer Science Education.

[B27-jintelligence-13-00157] Kraiger Kurt, Ford J. Kevin, Salas Eduardo (1993). Application of cognitive, skill-based, and affective theories of learning outcomes to new methods of training evaluation. Journal of Applied Psychology.

[B28-jintelligence-13-00157] Kuo Chih-Chen, Hou Huei-Tse (2025). Game-based collaborative decision-making training: A framework and behavior analysis for a remote collaborative decision-making skill training game using multidimensional scaffolding. Universal Access in the Information Society.

[B29-jintelligence-13-00157] Kurz Rainer, Bartram Dave (2002). Competency and Individual Performance: Modelling the World of Work. Organizational Effectiveness.

[B30-jintelligence-13-00157] Lai Xiaoyan, Wong Gary Ka-wai (2022). Collaborative versus individual problem solving in computational thinking through programming: A meta-analysis. British Journal of Educational Technology.

[B31-jintelligence-13-00157] Lai Xiaoyan, Ye Jiachu, Wong Gary Ka Wai (2023). Effectiveness of collaboration in developing computational thinking skills: A systematic review of social cognitive factors. Journal of Computer Assisted Learning.

[B32-jintelligence-13-00157] Lee Sang Joon, Francom Gregory M., Nuatomue Jeremiah (2022). Computer science education and K-12 students’ computational thinking: A systematic review. International Journal of Educational Research.

[B33-jintelligence-13-00157] Li Feng, Wang Xi, He Xiaona, Cheng Liang, Wang Yiyu (2022). The effectiveness of unplugged activities and programming exercises in computational thinking education: A Meta-analysis. Education and Information Technologies.

[B34-jintelligence-13-00157] Li Xinlei, Sang Guoyuan, Valcke Martin, van Braak Johan (2024). Computational thinking integrated into the English language curriculum in primary education: A systematic review. Education and Information Technologies.

[B35-jintelligence-13-00157] Li Yeping, Schoenfeld Alan H., diSessa Andrea A., Graesser Arthur C., Benson Lisa C., English Lyn D., Duschl Richard A. (2020). On Computational Thinking and STEM Education. Journal for STEM Education Research.

[B36-jintelligence-13-00157] Lin Yu-Shan, Chen Shih-Yeh, Tsai Chia-Wei, Lai Ying-Hsun (2021). Exploring Computational Thinking Skills Training Through Augmented Reality and AIoT Learning. Frontiers in Psychology.

[B37-jintelligence-13-00157] Lu Zhuotao, Chiu Ming M., Cui Yunhuo, Mao Weijie, Lei Hao (2022). Effects of Game-Based Learning on Students’ Computational Thinking: A Meta-Analysis. Journal of Educational Computing Research.

[B38-jintelligence-13-00157] Martins de Souza Adriano, Puglieri Fabio Neves, de Francisco Antonio Carlos (2024). Competitive Advantages of Sustainable Startups: Systematic Literature Review and Future Research Directions. Sustainability.

[B39-jintelligence-13-00157] Menolli André, Neto João Coelho (2022). Computational thinking in computer science teacher training courses in Brazil: A survey and a research roadmap. Education and Information Technologies.

[B40-jintelligence-13-00157] Merino-Armero José Miguel, José Antonio González-Calero, Cózar-Gutiérrez Ramón (2022). Computational thinking in K-12 education. An insight through meta-analysis. Journal of Research on Technology in Education.

[B41-jintelligence-13-00157] Page Matthew J., McKenzie Joanne E., Bossuyt Patrick M., Boutron Isabelle, Hoffmann Tammy C., Mulrow Cynthia D., Shamseer Larissa, Tetzlaff Jennifer M., Akl Elie A., Brennan Sue E. (2021). The PRISMA 2020 statement: An updated guideline for reporting systematic reviews. BMJ.

[B42-jintelligence-13-00157] Paleenud Inthira, Tanprasert Krittika, Waleeittipat Sakulkarn (2024). Lecture-Based and Project-Based Approaches to Instruction, Classroom Learning Environment, and Deep Learning. European Journal of Educational Research.

[B43-jintelligence-13-00157] Papert Seymour (1980). Mindstorms: Children, Computers, and Powerful Ideas.

[B44-jintelligence-13-00157] Passey Don (2017). Computer science (CS) in the compulsory education curriculum: Implications for future research. Education and Information Technologies.

[B45-jintelligence-13-00157] Pastor Miguel Antonio Soplin, Cervantes-Marreros Melany Dayana, Cubas-Pérez José Dilmer, Reategui-Apagueño Luis Alfredo, Tito-Pezo David, Piña-Rimarachi Jhim Max, Vasquez-Perez Cesar Adolfo, Correa-Vasquez Claudio Leandro, Rios Jose Antonio Soplin, del Pino Lisveth Flores (2025). Project-Based Learning at Universities: A Sustainable Approach to Renewable Energy in Latin America—A Case Study. Sustainability.

[B46-jintelligence-13-00157] Rao Toluchuri Shalini Shanker, Bhagat Kaushal Kumar (2024). Computational thinking for the digital age: A systematic review of tools, pedagogical strategies, and assessment practices. Educational Technology Research and Development.

[B47-jintelligence-13-00157] R Core Team (2021). R: A Language and Environment for Statistical Computing.

[B48-jintelligence-13-00157] Román-González Marcos, Pérez-González Juan-Carlos, Moreno-León Jesús, Robles Gregorio (2018). Extending the nomological network of computational thinking with non-cognitive factors. Computers in Human Behavior.

[B49-jintelligence-13-00157] Samdrup Tshering, Fogarty James, Pandit Ram, Iftekhar Md Sayed, Dorjee Kinlay (2023). Does FDI in agriculture in developing countries promote food security? Evidence from meta-regression analysis. Economic Analysis and Policy.

[B50-jintelligence-13-00157] Shea Beverley J., Reeves Barnaby C., Wells George, Thuku Micere, Hamel Candyce, Moran Julian, Moher David, Tugwell Peter, Welch Vivian, Kristjansson Elizabeth (2017). AMSTAR 2: A critical appraisal tool for systematic reviews that include randomised or non-randomised studies of healthcare interventions, or both. BMJ.

[B51-jintelligence-13-00157] Sun Dan, Looi Chee-Kit, Li Yan, Zhu Chengcong, Zhu Caifeng, Cheng Miaoting (2024). Block-based versus text-based programming: A comparison of learners’ programming behaviors, computational thinking skills and attitudes toward programming. Educational Technology Research and Development.

[B52-jintelligence-13-00157] Sun Lihui, Zhou Liang (2023). Does text-based programming improve K-12 students’ CT skills? Evidence from a meta-analysis and synthesis of qualitative data in educational contexts. Thinking Skills and Creativity.

[B53-jintelligence-13-00157] Sun Lihui, Zhen Guo, Hu Linlin (2023). Educational games promote the development of students’ computational thinking: A meta-analytic review. Interactive Learning Environments.

[B54-jintelligence-13-00157] Sun Lihui, Hu Linlin, Zhou Danhua (2021). Which way of design programming activities is more effective to promote K-12 students’ computational thinking skills? A meta-analysis. Journal of Computer Assisted Learning.

[B55-jintelligence-13-00157] Tang Xiaodan, Yin Yue, Lin Qiao, Hadad Roxana, Zhai Xiaoming (2020). Assessing computational thinking: A systematic review of empirical studies. Computers & Education.

[B56-jintelligence-13-00157] Threekunprapa Arinchaya, Yasri Pratchayapong (2021). The role of augmented reality-based unplugged computer programming approach in the effectiveness of computational thinking. International Journal of Mobile Learning and Organisation.

[B57-jintelligence-13-00157] Tikva Christina, Tambouris Efthimios (2021a). A systematic mapping study on teaching and learning Computational Thinking through programming in higher education. Thinking Skills and Creativity.

[B58-jintelligence-13-00157] Tikva Christina, Tambouris Efthimios (2021b). Mapping computational thinking through programming in K-12 education: A conceptual model based on a systematic literature Review. Computers & Education.

[B59-jintelligence-13-00157] Vakhabova Selima Aslambekovna, Kosulin Valery V., Zizaeva Ana (2025). Artificial intelligence in education: Challenges and opportunities for sustainable development. Ekonomika i Upravlenie: Problemy, Resheniya.

[B60-jintelligence-13-00157] Valls Pou Albert, Canaleta Xavi, Fonseca David (2022). Computational Thinking and Educational Robotics Integrated into Project-Based Learning. Sensors.

[B61-jintelligence-13-00157] Viechtbauer Wolfgang (2010). Conducting Meta-Analyses in R with the metafor Package. Journal of Statistical Software.

[B62-jintelligence-13-00157] Wang Xiaowen, Chan Kan Kan, Li Qianru, Leung Shing On (2024). Do 3–8 Years Old Children Benefit From Computational Thinking Development? A Meta-Analysis. Journal of Educational Computing Research.

[B63-jintelligence-13-00157] Wang Xinyue, Cheng Mengmeng, Li Xinfeng (2023). Teaching and Learning Computational Thinking Through Game-Based Learning: A Systematic Review. Journal of Educational Computing Research.

[B64-jintelligence-13-00157] Wang Yang, Xie Bin (2024). Can robot-supported learning enhance computational thinking?—A meta-analysis. Thinking Skills and Creativity.

[B65-jintelligence-13-00157] Weng Xiaojing, Ye Huiyan, Dai Yun, Ng Oi-lam (2024). Integrating Artificial Intelligence and Computational Thinking in Educational Contexts: A Systematic Review of Instructional Design and Student Learning Outcomes. Journal of Educational Computing Research.

[B66-jintelligence-13-00157] Wing Jeannette M. (2006). Computational thinking. Communications of the ACM.

[B67-jintelligence-13-00157] Wohlfart Olivia, Wagner Ingo (2023). Teachers’ role in digitalizing education: An umbrella review. Educational Technology Research and Development.

[B68-jintelligence-13-00157] Wongwatkit Charoenchai, Panjaburee Patcharin, Srisawasdi Niwat, Seprum Pongpon (2020). Moderating effects of gender differences on the relationships between perceived learning support, intention to use, and learning performance in a personalized e-learning. Journal of Computers in Education.

[B69-jintelligence-13-00157] Wu Ting-Ting, Silitonga Lusia Maryani, Murti Astrid Tiara (2024). Enhancing English writing and higher-order thinking skills through computational thinking. Computers & Education.

[B70-jintelligence-13-00157] Xu Enwei, Wang Wei, Wang Qingxia (2023). A meta-analysis of the effectiveness of programming teaching in promoting K-12 students’ computational thinking. Education and Information Technologies.

[B71-jintelligence-13-00157] Ye Huiyan, Liang Biyao, Ng Oi-Lam, Chai Ching Sing (2023). Integration of computational thinking in K-12 mathematics education: A systematic review on CT-based mathematics instruction and student learning. International Journal of STEM Education.

[B72-jintelligence-13-00157] Yeni Sabiha, Grgurina Nataša, Saeli Mara, Hermans Felienne, Tolboom Jos, Barendsen Erik (2024). Interdisciplinary integration of computational thinking in K-12 education: A systematic review. Informatics in Education.

[B73-jintelligence-13-00157] Yin Stella Xin, Goh Dion Hoe-Lian, Quek Choon Lang (2024). Collaborative Learning in K-12 Computational Thinking Education: A Systematic Review. Journal of Educational Computing Research.

[B74-jintelligence-13-00157] Zhang LeChen, Nouri Jalal (2019). A systematic review of learning computational thinking through Scratch in K-9. Computers & Education.

[B75-jintelligence-13-00157] Zhang Wuwen, Guan Yurong, Hu Zhihua (2024a). The efficacy of project-based learning in enhancing computational thinking among students: A meta-analysis of 31 experiments and quasi-experiments. Education and Information Technologies.

[B76-jintelligence-13-00157] Zhang Yanjun, Luo Ronghua, Zhu Yijin, Yin Yuan (2021). Educational Robots Improve K-12 Students’ Computational Thinking and STEM Attitudes: Systematic Review. Journal of Educational Computing Research.

[B77-jintelligence-13-00157] Zhang Yanjun, Liang Yanping, Tian Xiaohong, Yu Xiao (2024b). The effects of unplugged programming activities on K-9 students’ computational thinking: Meta-analysis. Educational Technology Research and Development.

